# Supplementary material for: Towards passive non-line-of-sight acoustic localization around corners using uncontrolled random noise sources
Source: Sci Rep. 2023 Mar 27;13:4952. doi: 10.1038/s41598-023-31490-2 (PMC10043274; doi:10.1038/s41598-023-31490-2)
Supplement: Supplementary file 1 — Supplementary Figures. [file 41598_2023_31490_MOESM1_ESM.pdf]

Towards Passive Non-Line-of-Sight Acoustic Localization Around Corners Using Uncontrolled Random Noise Sources
Supplementary materials

Jeremy Boger-Lombard[1], Yevgeny Slobodkin[1] and Ori Katz[1*]

[1]Applied Physics Department, The Hebrew University of Jerusalem,
Jerusalem, 9190401, Israel

*orik@mail.huji.ac.il

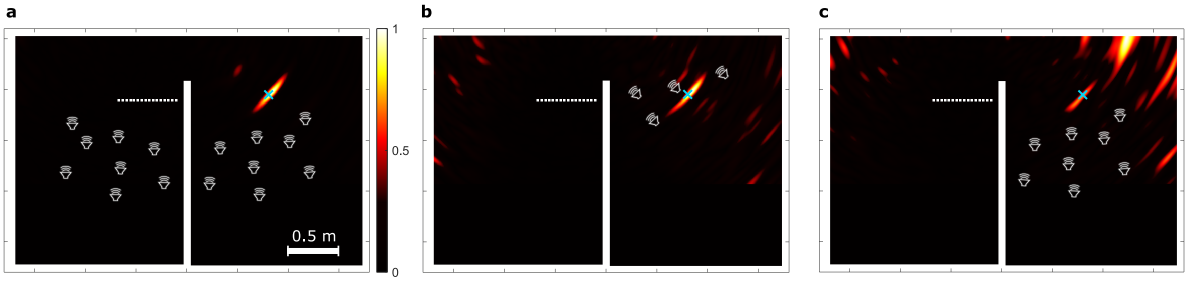

Supplementary Figure 1. Numerical study of sources locations effect on NLoS localization. 16 detectors (marked by white dots) are placed in a reverberating rectangular room with different number and positions of random uncorrelated broadband sources. The reconstructions are mirrored with respect to the top wall. A cyan cross marks the true position of the target. **a** 16 sources positioned on both sides of the barrier. **b,c** Scenes where all sources are positioned around the barrier on the same side as the object, with 4 directional sources (b) and 8 omnidirectional sources(c).

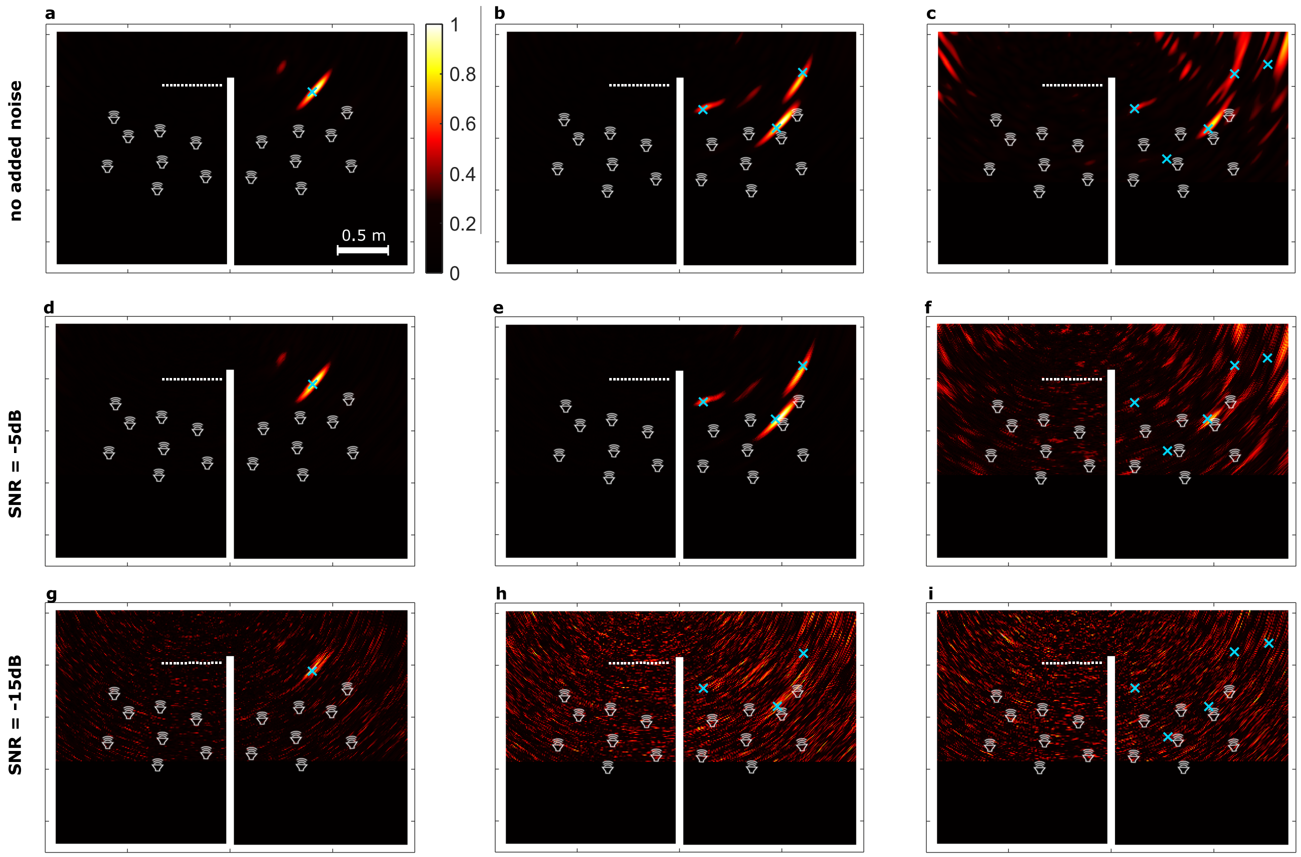

Supplementary Figure 2. Numerical investigation of reconstruction fidelity under different signal to noise ratios (SNR) and for different number of targets (scene complexity). The considered scenes are composed of either one target **a,d,g**, three targets **b,e,h**, and five targets **c,f,i**. (a-c) no added noise. **d-f** same as (a-c) with added detector noise providing an $SNR = -5dB$. **g-i** same as (d-f) with $SNR = -15dB$. The reconstructions are mirrored with respect to the top wall. Cyan crosses mark the true positions.
